# Helminth infection–induced malignancy

**DOI:** 10.1371/journal.ppat.1006393

**Published:** 2017-07-27

**Authors:** Paul J. Brindley, Alex Loukas

**Affiliations:** 1 Department of Microbiology, Immunology and Tropical Medicine, School of Medicine & Health Sciences, The George Washington University, Washington DC, United States of America; 2 Research Center for Neglected Tropical Diseases of Poverty, School of Medicine & Health Sciences, The George Washington University, Washington DC, United States of America; 3 Centre for Biodiscovery and Molecular Development of Therapeutics, Australian Institute of Tropical Health & Medicine, James Cook University, Cairns, Queensland, Australia; University of Wisconsin Medical School, UNITED STATES

## Infection with some helminth pathogens represents a biological carcinogen

Infectious diseases cause more than 20% of cancers in the developing world [1]. About a dozen pathogens including Epstein-Barr virus and human T cell lymphocytotropic virus 1 are among the well-known examples. In addition, infection with several trematodes, which are eukaryotes, can cause malignancy. The International Agency for Research on Cancer categorizes infection with the fish-borne trematodes *Opisthorchis viverrini* and *Clonorchis sinensis* and the blood fluke *Schistosoma haematobium* as Group 1 biological carcinogens [2]. In addition to parasitism directly damaging development, health, and prosperity of infected populations, infection with these helminths leads to cholangiocarcinoma (CCA) (bile duct cancer) and squamous cell carcinoma (SCC) of the urinary bladder, respectively [2]. By contrast, infection with phylogenetic relatives, also trematodes of the phylum Platyhelminthes and also major pathogens, is not carcinogenic. These irregularities suggest that either helminth-specific metabolites contribute to tumorigenesis and/or that certain tissues or organs are particularly susceptible to infection-induced malignancy. Moreover, each of these helminth infections must be viewed holistically in the context of a perfect storm of risk for cancer (see [3]).

## Helminth infection–induced cancers

### *O*. *viverrini* and *C*. *sinensis* infection–induced cholangiocarcinoma

**Fig 1:**
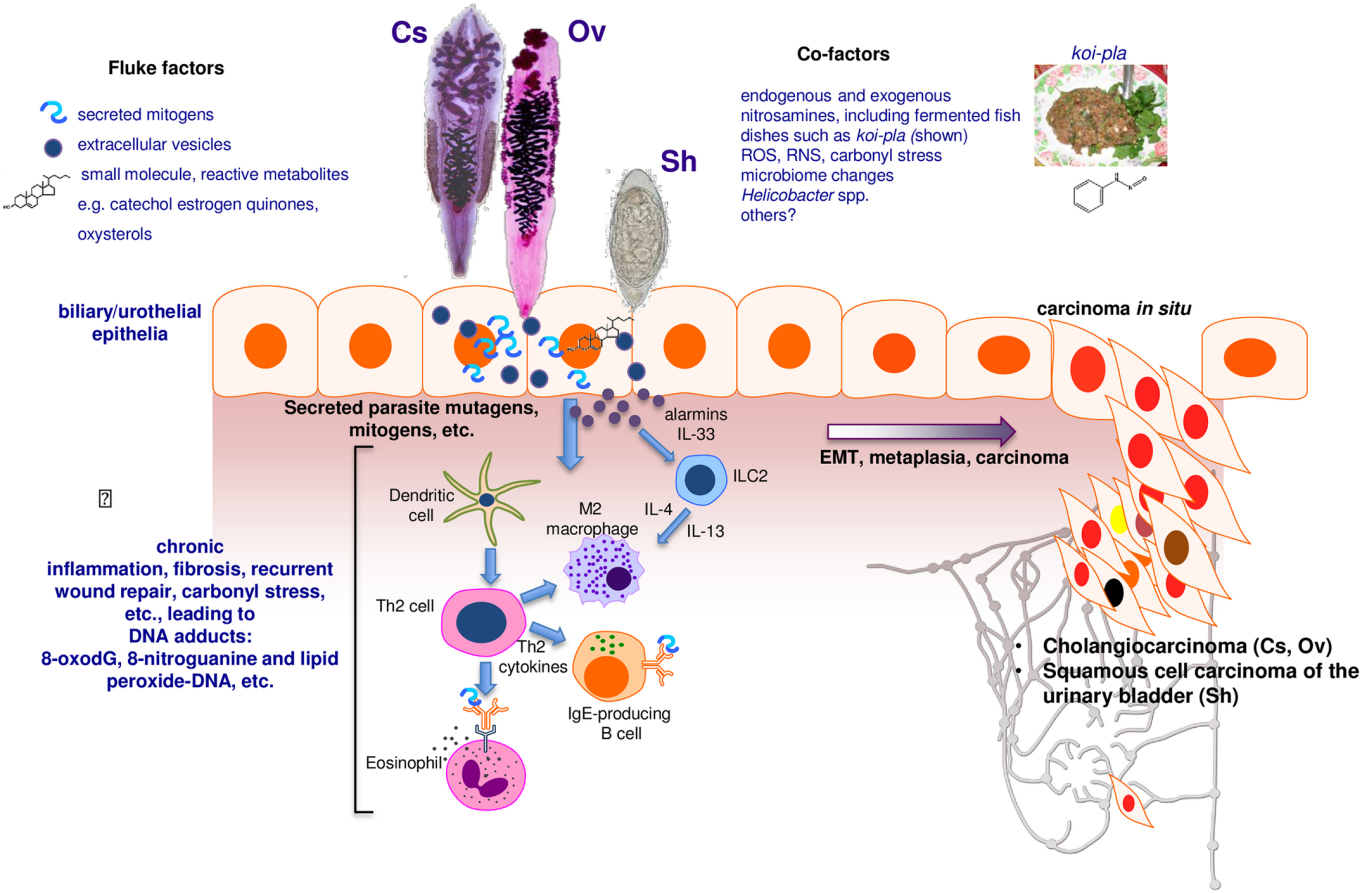
Schematic representation of hypothesized processes of carcinogenesis of the biliary tract and urinary bladder during chronic infection with fish-borne liver flukes *Opisthorchis viverrini* (Ov) and *Clonorchis sinensis* (Cs) and the blood fluke *Schistosoma haematobium* (Sh). Photomicrographs: adult developmental stages of Cs and Ov and the egg stage of Sh. During infection, mutations initiate carcinogenesis, perhaps as the consequence of interaction of epithelial cell chromosomal DNA with inflammation-associated reactive oxygen species (ROS) and reactive nitrogen species (RNS) and lipid peroxidation and/or metabolites released by the worms, such as catechol estrogen quinones and oxysterols. Subsequently, mediators of helminth origin such as granulin from Ov promote epidermal to mesenchymal transition (EMT), transformed cell growth, complementary angiogenesis, down-regulation of apoptosis, and other hallmarks of cancer.

Infection is accomplished by ingestion of undercooked freshwater fish infected with the metacercaria stage of these species of liver flukes. Human infection leads to hepatobiliary disease, including cholangitis and periductal fibrosis. In liver fluke–endemic regions, infection is the major risk factor for CCA [2, 4, 5]. CCAs are slow-growing adenocarcinomas that metastasize to distant sites due to proximity to lymphatic vessels. Liver fluke–related CCA is often diagnosed at an advanced stage, when the primary cancer is no longer amenable to curative surgery [5]. The mechanism(s) by which infection initiates genetic lesions that eventually culminate in CCA is not understood, although it is likely to be multifactorial, involving biliary tract and systemic chronic inflammation and associated endogenous nitrosation [6, 7], secretion of mitogens and other mediators by the parasite, and cofactors including dietary preferences for nitrosamines-rich foods [4, 8, 9] (Fig 1).

### Urogenital schistosomiasis and bladder cancer

Three major species of schistosomes are the agents of schistosomiasis: *S*. *japonicum* and *S*. *mansoni* cause intestinal schistosomiasis whereas *S*. *haematobium* causes urogenital schistosomiasis. Of about 112 million cases of *S*. *haematobium* infection in sub-Saharan Africa, 70 million are associated with hematuria, 18 million with major bladder wall pathology, and 10 million with hydronephrosis leading to kidney damage. Deposition of ova of *S*. *haematobium* in the bladder wall can lead to SCC of the bladder [2, 10].

## Initiation, promotion, and progression of tumorigenesis

Where opisthorchiasis is endemic, people can remain infected for decades. Opisthorchiasis provokes inflammation of the biliary tree, with hyperplasia and metaplasia of the cholangiocytes that line the biliary tract adjacent to the flukes. Opisthorchiasis-induced fibrosis engulfs the proliferating cells, manifesting as biliary periductal fibrosis [4, 11]. Chronic inflammation in response to parasite metabolites and growth factors is implicated in the inflammatory response linked with infection [11]. Additional factors including carriage of *Helicobacter* and other microbiome changes within the biliary tract might participate [8, 12]. Elevated plasma interleukin-6 (IL-6) is associated with marked increase in risk of periductal fibrosis during opisthorchiasis and compounds pathogenesis by promoting a fibrogenic inflammatory milieu. The cell of origin of CCA is the cholangiocyte, a specialized epithelial cell that lines the bile duct. Following initiation, for example by oxidation of cholangiocyte chromosomal DNA by oxysterols generated by reactive oxygen species (ROS) and reactive nitrogen species (RNS) arising during opisthorchiasis-induced oxidative stress [13] and/or oxysterols released by the liver fluke [8, 14], oncogenesis appears to be promoted by cholestasis and chronic inflammation. The release and downstream consequences of IL-6, platelet-derived growth factor, tumor necrosis factor-alpha (TNF-α), and transforming growth factor-beta (TGF-β) are pivotal to the proliferation of cholangiocytes. Autonomous proliferation, evasion of apoptosis, and angiogenesis sustain the incipient neoplasm [11].

Bladder cancer is the most common tumor of the urinary system and consists of 2 main forms—urothelial carcinoma (UCC) and SCC. The bladder is lined by a specialized epithelium termed the urothelium, which is exposed routinely to potential carcinogens and hence is at particular risk of cancer. The urothelium is a stratified epithelium composed of keratin 5 (K5)-expressing basal cells, intermediate cells, and umbrella cells. K5-positive basal cells likely give rise to SCC [10, 15–17]. UCC accounts for around 90% of bladder cancers, and major risk factors include tobacco smoking, occupational exposure to aromatic amines and polycyclic hydrocarbons, and bladder stones, among others [10, 15]. In regions endemic for urogenital schistosomiasis, SCC is more common than UCC and may be the cancer of highest incidence [2, 10].

Schistosome eggs entrapped in the bladder wall release metabolites, presumably to facilitate egress of the egg to the lumen and to the external environment. The process leads to hematuria and to chronic inflammation, in turn increasing the risk of urothelial hyperplasia, dysplasia, and SCC (Fig 1). Urogenital schistosomiasis (UGS) is a chronic infection, often interrupted by drug treatment and often followed in turn by reinfection. Fibrosis induced by entrapped schistosome eggs may promote cellular proliferation, hyperplasia, and metaplasia that eventually induce carcinogenesis [10]. Mass spectrometric analysis of urine during UGS reveals estrogen-like metabolites including catechol estrogen quinones (CEQ), likely of schistosome origin, CEQ-DNA adducts, and novel metabolites derived from 8-oxo-7, 8-dihydro-2′-deoxyguanosine (8-oxodG) [18]. Nitrosamines and increased levels of beta-glucuronidase and cyclooxygenase-2 derived from schistosomes also represent potential bladder carcinogens. These helminth infection–derived carcinogens may damage DNA, leading to somatic mutations in oncogenes such as *p53*, retinoblastoma protein, epidermal growth factor receptor, and erbB2 receptor tyrosine kinase. In like fashion, chromosomal adducts including 8-oxodG and 8-nitroguanine and lipid peroxide-DNA increase during opisthorchiasis (see [19]).

Investigation of UGS-induced bladder cancer is challenging given that rodent models do not exhibit urogenital disease; infection with *S*. *haematobium* causes hepatointestinal disease in rodents. However, in a recently developed rodent model, eggs of *S*. *haematobium* injected into the bladder wall of mice provoke egg-associated pathogenesis more reflective of the human condition [20, 21]. Preneoplastic lesions involving epithelial to mesenchymal (EMT)-like profiles have been described in this model following coadministration of exogenous nitrosamines [22]. These approaches might lead to deeper understanding of the carcinogenesis of UGS-induced SCC. A hamster model involving coadministration of nitroso compounds and liver fluke infection has long been employed to study infection-induced bile duct cancer [2, 4, 11, 19, 23]. Curiously, among the human schistosomes, only infection with *S*. *haematobium* is categorized as a biological carcinogen [2]. Perhaps local levels of metabolites such as CEQ are not produced by *S*. *mansoni* and *S*. *japonicum* and/or the hepatointestinal niche of these intestinal disease–causing schistosomes is less disposed to schistosome infection–induced malignancy.

## Mutations, mutational signatures, rearrangements, epigenetics

Cancer arises when mutations occur in the DNA of the genome of the target cell and the mutations lead to uncontrolled cellular proliferation, invasion, and metastasis. The landscape of mutations that accumulate within the tumor record the mutagenic processes that have taken place over the life span of the malignancy. Each mutation endows an imprint on the genome of the tumor, documenting the types of DNA lesion and repair processes that lead to base substitutions, insertions, deletions, and structural variations. These mutational profiles have implications for diagnosis, therapy, and public health interventions.

Metabolites of helminth origin, including oxysterols, catechol estrogens, heme from ingested blood, and others, all of which are reactive species, may depurinate host cell DNA, leading to error-prone repair that results in mutations of cancer driver genes [8, 18]. At the genomic level, analysis of the mutation profiles of *O*. *viverrini*–related versus nonliver fluke–induced CCA reveals marked variation in mutation patterns [24]. Somatic mutations occur frequently in the tumor suppressor genes *p53* and *smad4* in *O*. *viverrini*–induced CCA. By contrast, somatic mutations in the genes encoding BRCA1 associated protein-1 and isocitrate dehydrogenases 1 and 2 are more common in non-*O*. *viverrini*–associated CCA [24, 25]. Mutations in *p53* and *smad4* directly affect the p53- and TGF-signaling pathways, both of which are involved in tumorigenesis. Thus, distinct causes of CCA induce discrete somatic alterations, even within the same cancer type [25]. Emphasizing this point, liver fluke infection–induced CCA exhibits altered DNA methylation and transcriptional profiles reflective of xenobiotic metabolism and pro-inflammatory responses in comparison to nonliver fluke infection–induced CAA and to healthy biliary duct [24–26].

Details also have emerged from whole exome sequences of UCC, although the specific mutational landscape of SCC and hence UGS-induced SCC have not been reported [2, 15]. Mutation of sonic hedgehog can initiate carcinogenesis in bladder cancer [16]. Recurrent mutations occur in >30 other genes involved in cell proliferation, differentiation, genetic stability, and specifically cell-cycle regulation, chromatin regulation, and kinase signaling pathways [27]. Bladder cancer frequently exhibits C to T or C to G mutations at TC dinucleotides, which may reflect hyperactive DNA editing by apolipoprotein B mRNA-editing enzyme, catalytic polypeptide-like (APOBEC) cytidine deaminases [28].

## Host–parasite interactions

**Fig 2:**
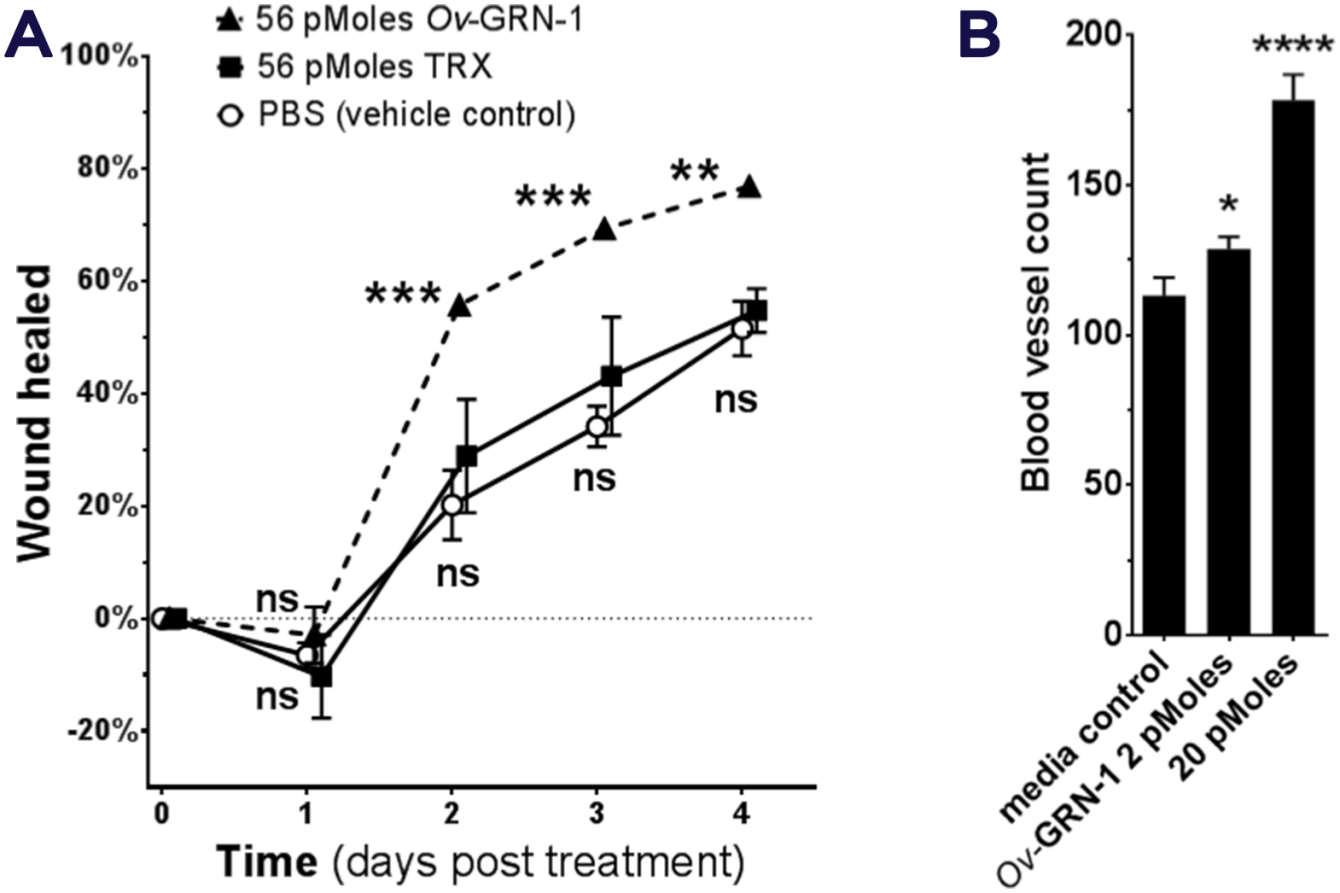
Liver fluke granulin promotes wound repair and angiogenesis. *Opisthorchis viverrini* secretes a growth factor termed liver fluke granulin, *Ov*-GRN-1, an orthologue of mammalian granulin. *Ov*-GRN-1 stimulates cholangiocytes to proliferate and promotes wound healing and angiogenesis. A recent report on *Ov*-GRN-1 [32] illustrates some of these carcinogenic-conducive properties of the growth factor, as follows: **(A)** rate of wound closure on a cutaneous lesion on mice, **(B)** the angiogenic nature of *Ov*-GRN-1 revealed using the chorioallantoic membrane assay in fertilized quail eggs.

Communication between helminths and host cells likely evolved to facilitate parasitism. Communicating metabolites may, however, contribute to carcinogenesis [8, 29]. For example, *O*. *viverrini* secretes a growth factor termed *Ov*-GRN-1 that shares homology with human granulin [30]. Secreted *Ov*-GRN-1 may promote angiogenesis and also wound repair of the bile ducts damaged by the liver fluke [11, 30] (Fig 2). Moreover, *O*v-GRN-1 may mimic the action of interleukin-33 (IL-33), an epithelial mitogen for cholangiocytes, in the development of CCA [31]. IL-33 primes type 2 innate lymphoid cells to induce proliferation of neighboring cholangiocytes by the release of Interleukin-13 (IL-13). Antibodies to *Ov*-GRN-1 blocked its ability to drive proliferation of host cells [30, 32]. Liver flukes also secrete extracellular vesicles (EVs) [33] (Fig 1). The membranes surrounding EVs are enriched in tetraspanins, proteins that interact with transmembrane and cytosolic signaling proteins [34]. Cholangiocytes internalize both *O*. *viverrini* EVs and *Ov*-GRN-1 [32]. Antibodies to a tetraspanin located on the surface of *O*. *viverrini* EVs block the internalization by cholangiocytes of liver fluke EVs and suppress the proliferation and secretion of IL-6 by cholangiocytes [33]. Other helminth proteins induce changes that reflect the hallmarks of cancer, including EMT phenotypes, pro-inflammatory cytokines, and repression of apoptosis [32, 35]. Type 2 T helper cell (Th2) responses induced by schistosome proteins including interleukin 4-inducing principle of schistosome eggs (IPSE) not only facilitate egress of the schistosome egg to the bladder lumen by modulating the granuloma but also appear to modulate vasculogenic and cellular responses conducive to neoplasia [10, 36]. Targeting these or other parasite–host cell communicating proteins with vaccines may not only block helminth infections but deliver novel anticancer vaccines [11].
